# First Report of a Complete Genome Sequence of White spot syndrome virus from India

**DOI:** 10.1128/genomeA.00055-18

**Published:** 2018-02-22

**Authors:** K. Vinaya Kumar, M. S. Shekhar, S. K. Otta, K. Karthic, J. Ashok Kumar, G. Gopikrishna, K. K. Vijayan

**Affiliations:** a Nutrition Genetics and Biotechnology Division, ICAR-Central Institute of Brackishwater Aquaculture, Chennai, Tamil Nadu, India; b Aquatic Animal Health and Environment Division, ICAR-Central Institute of Brackishwater Aquaculture, Chennai, Tamil Nadu, India

## Abstract

White spot syndrome virus is a major pathogen of shrimp, causing economic loss to the aquaculture industry. For the first time, a complete *de novo* genome of an Indian isolate of this virus has been deciphered using Illumina and Nanopore sequencing technologies. The genome has 280,591 bp with 442 predicted coding genes.

## GENOME ANNOUNCEMENT

White spot syndrome virus (WSSV) is the most devastating viral pathogen of shrimp, causing up to 100% cumulative mortality within 3 to 7 days (1). The WSSV isolate IN_AP4RU was obtained from the pleopods of infected Pacific white shrimp (Penaeus vannamei) samples that were collected from a shrimp culture pond located in Andhra Pradesh State, India, in 2014. The presence of the virus was confirmed through a PCR using specific primers reported for the detection of WSSV (2).

Two sequencing technologies were used to build the complete genome of this virus. The assembly was principally built with long Nanopore reads, which were corrected for error bases using short Illumina reads. The genomic DNA extracted from WSSV-infected shrimp pleopods was used to prepare an Illumina-compatible library with a NEXTflex rapid DNA sequencing kit (BIOO Scientific, Austin, TX, USA). Sequencing on the Illumina NextSeq500 platform generated 14,138,233 (2 × 150-bp) paired-end reads. The same DNA was used to prepare a Nanopore-compatible library, which was sequenced on the MinION Mk1b platform (Oxford Nanopore Technologies, Oxford, UK) using a SpotON flow cell (FLO-MIN106), MinKNOW version 1.10.11, and the Albacore version 2.1.3 base caller in a 48-h sequencing protocol to generate 170,475 reads. The average, median, and *N*_50_ lengths of the raw Nanopore reads were 920.2 bp, 7,978 bp, and 1,186 bp, respectively. The Illumina and Nanopore reads were separately aligned to a WSSV reference genome (GenBank accession no. KT995472) using the Burrows-Wheeler alignment tool to isolate virus-specific DNA reads. About 771,852 Illumina paired-end reads were used to correct 27,883 Nanopore reads that originated from viral DNA.

The error-corrected Nanopore reads, which had total and *N*_50_ lengths of 31,937,652 bp and 2,049 bp, respectively, and more than 100× coverage, were used for *de novo* assembly using Canu version 1.1. The assembly generated a single contig of 280,591 bp. Although akin to a Chinese WSSV isolate of 281,054 bp (3), this is the smallest genome reported so far for WSSV. The genome fragment was analyzed with the Rapid Annotations using Subsystems Technology (RAST) server (4), specifying the “Virus” domain, which predicted about 442 coding sequences. The annotation of the coding sequences was obtained through a local BLAST search against the UniProt Virus database using the Blast2Go tool with an E value of 1E−5. The complete genome sequence of the Indian isolate of WSSV obtained in this study will help in understanding its genome composition and the variability among other geographical isolates of WSSV.

### Accession number(s).

The complete genome sequence reported here has been submitted to NCBI/GenBank under the accession no. MG702567.
